# Chemical Characteristics of Electron Shuttles Affect Extracellular Electron Transfer: *Shewanella decolorationis* NTOU1 Simultaneously Exploiting Acetate and Mediators

**DOI:** 10.3389/fmicb.2019.00399

**Published:** 2019-03-05

**Authors:** Shiue-Lin Li, Yu-Jie Wang, Yu-Chun Chen, Shiu-Mei Liu, Chang-Ping Yu

**Affiliations:** ^1^Graduate Institute of Environmental Engineering, National Taiwan University, Taipei, Taiwan; ^2^Institute of Marine Biology, National Taiwan Ocean University, Keelung, Taiwan

**Keywords:** extracellular electron transfer, electron-balance calculation, *Shewanella decolorationis* NTOU1, electron shuttle mediator, outer membrane cytochrome

## Abstract

In the present study, we found that our isolate *Shewanella decolorationis* NTOU1 is able to degrade acetate under anaerobic condition with concomitant implementation of extracellular electron transfer (EET). With +0.63 V (vs. SHE) poised on the anode, in a 72-h experiment digesting acetate, only 2 mM acetate was consumed, which provides 6% of the electron equivalents derived from the initial substrate mass to support biomass (5%) and current generation (1%). To clarify the effects on EET of the addition of electron-shuttles, riboflavin, anthraquinone-2,6-disulfonate (AQDS), hexaammineruthenium, and hexacyanoferrate were selected to be spiked into the electrochemical cell in four individual experiments. It was found that the mediators with proton-associated characteristics (i.e., riboflavin and AQDS) would not enhance current generation, but the metal-complex mediators (i.e., hexaammineruthenium, and hexacyanoferrate) significantly enhanced current generation as the concentration increased. According to the results of electrochemical analyses, the *i*-*V* graphs represent that the catalytic current induced by the primitive electron shuttles started at the onset potential of −0.27 V and continued increasing until +0.73 V. In the riboflavin-addition experiment, the catalytic current initiated at the same potential but rapid saturated beyond −0.07 V; this indicated that the addition of riboflavin affects mediator secretion by *S. decolorationis* NTOU1. It was also found that the current was eliminated after adding 48 mM *N*-acetyl-L-methionine (i.e., the cytochrome inhibitor) when using acetate as a substrate, indicating the importance of outer-membrane cytochrome.

## Introduction

Bioelectrochemical systems (BESs) can now be derived as technologies or applications that utilize the electrochemical interaction of microbes and electrodes, which is usually powered by oxidizing organic-matter oxidation by means of the redox reactions of microorganisms (or the other biological moieties like enzyme and cell organelle) occurring on the anode (Schroder et al., 2015). To enhance the performances of the anode, the most promising way is to facilitate extracellular electron transfer (EET) either by selectively inoculating EET capable microorganisms, or adding fixed [e.g., tungsten carbide (Rosenbaum et al., 2006) and α-Fe_2_O_3_ (Nakamura et al., 2009)] or diffusive electron shuttles in proximity to the electrodes to chemically assist the EET. Diffusive electron shuttles used as mediators need some essential properties, including high diffusion coefficients, rapid electron transfer, sustainability in repeated redox turnover, and non-cytotoxicity (Bullen et al., 2006). The soluble electron shuttles usually work as transferring across the porin to the cell interior (Ikeda, 2012) in order to bring the electrons out from the internal redox protein (e.g., nicotinamide adenine dinucleotide dehydrogenase, NDH, Li et al., 2017) to the outer electron acceptors, or directly exchange electrons with the outer membrane cytochromes (OMCs, Coursolle et al., 2010). Moreover, recent studies indicated that electron shuttles (i.e., flavin mononucleotide and riboflavin) might interact with OMC by direct bonding, creating the shortest physical distance to favor the electron flow (Okamoto et al., 2013). In addition, in our recent study, we reported that tricarboxylic-acid (TCA)-cycle activities stopped due to excessive mediator addition. This result indicates that *Shewanella* spp. cannot obtain the necessary adenosine triphosphate (ATPs) via the oxidative phosphorylation at high mediator concentrations (Li et al., 2018).

Diffusive electron shuttles were frequently found in the wastewater or groundwater of an aquifer; this will be the electrolyte for MFC development in the future. For example, a large amount of riboflavin is found in the wastewater of food or pharmaceutical industries (Qian et al., 2009; Sun et al., 2013). Dyes like safranine and methylene blue may be found in the wastewater discharged by the industries which need to color their products (Gupta et al., 2011). These have been used as an effective electron shuttle in the MFC study (Choi et al., 2003; Miroliaei et al., 2015). The humic substances (typically studied using anthraquinone-2,6-disulfonate (AQDS) as a model substitution, Newman and Kolter, 2000) and ferrocyanide (formed in a nuclear-waste-processing site, Plymale et al., 2018) possess redox capabilities and exist in the ground water. Therefore, regarding scaling-up BES for practical applications, it is of utmost importance—essential, in fact—to know just how these external mediators affect EET.

Microorganisms require different metabolic pathways to deal with different substrates. Without competitive electron acceptors like sulfate and nitrate on the anode, for example, glucose may be fermented into acetate and butyrate that absorb two-thirds of substrate electrons, with a resultant low electron recovery (Rabaey and Verstraete, 2005). When lactate is used as the substrate, the metabolism diverging from acetyl-CoA can either enter the TCA cycle or can be reversibly transferred into acetate production to generate ATP (i.e., substrate-level phosphorylation). As for acetate, the only metabolism to extract energy from it is by implementing the TCA cycle for completed oxidation. While the *Shewanella* spp. have been intensively applied in many BES studies so far (Li et al., 2010; Zhang et al., 2017; Li et al., 2018), to our best knowledge, no reported study has yet to utilize acetate as an electron donor to drive EET on the *Shewanella* anodes, although it was reported that *Shewanella* spp. are able to use acetate to reduce some electron acceptors, such as nitrate, which can be uptaken without the need of EET (Yoon et al., 2013). Surprisingly, in our preliminary study, we found that our isolate *Shewanella decolorationis* NTOU1 was able to use acetate for EET without oxygen. To clarify the interaction between the central metabolism and phosphorylation during EET of *Shewanella* sp., it is interesting to test the current generation in an electrochemical cell using different substrates that reveals different pathways of metabolism and phosphorylation.

The objective of the present study is to clarify the effect of mediator addition on EET by *S. decolorationis* NTOU1, especially when acetate is the sole electron donor on the anode. First, the concept of electron-balance calculation was employed to understand the electron distribution when *S. decolorationis* NTOU1 was exploiting different substrates (i.e., lactate and acetate) for current generation. To study the effect of electron-shuttle addition, four different mediators were used to treat the *S. decolorationis* NTOU1 anode (connected to a potentiostat) via a serial spiking that generates an array of mediator concentrations in one experimental run: riboflavin; AQDS; hexaammineruthenium (III) chloride (Ru(NH_3_)_6_^3+^); and potassium hexacyanoferrate (Fe(CN)_6_^3−^). By analyzing the *i*-*V* graph, it could be clarified whether the externally added mediators could be a useful addition, or if they may inhibit the primitive EET mechanisms of *S. decolorationis* NTOU1. Finally, the experiment involving the addition of a cytochrome inhibitor (i.e., *N*-acetyl-L-methionine, AcMet) addition can clarify the importance of OMCs to EET.

## Materials and Methods

### Microorganism and Cultural Condition

Detailed information on *S. decolorationis* NTOU1 is given elsewhere (Li et al., 2010). The strains of *S. oneidensis* MR-1 and *S. putrefaciens* ATCC8071 were purchased from Bioresource Collection and Research Center, Hsinchu, Taiwan. Prior to the electrochemical experiments, both strains were pre-cultured using the Luria-Bertani medium for 24 h at 30°C, harvested by centrifugation (4,629 g, 5 min, 26°C), and then washed three times with a neutral pH phosphate buffer (0.1 M, pH 7) prior to inoculation, to avoid bringing in any unexpected mediators left in the cultural media (Masuda et al., 2010).

### Configuration and Medium of the Bioelectrochemical Cells

In this study, two-chamber bioelectrochemical cells consisting of a working-electrode (anode) chamber and a counter-electrode (cathode) chamber were used to evaluate the efficiency of current generation. The inner walls of 200-mL anode chambers were lined with 24 cm^2^ of carbon felt which was secured using a Teflon spacer. For the alternative electron donor experiment described in section 3.1, 0.75-mm-thick carbon felt was used (B0050, Toray, Japan). In the other experiments, the thick carbon felt was manually sectioned into a thickness of 3 mm (1-cm-thick, Gansu Haoshi Carbon Fiber Co., Ltd., China) for the following usage. The amount of initial inoculated biomass was about 60 mg-cell L^−1^ in each bioelectrochemical cell as harvested from 10 mL of the pre-culture broth; *t* = 0 h in all Figures indicates the moment *S. decolorationis* NTOU1 was inoculated. In all experiments, the working electrode potential was controlled at +0.63 V (vs. SHE) using a potentiostat (HA-151A, Hokuto Denko, Tokyo, Japan). The response current was recorded using a data acquisition system (GL240, Graphtec, Yokohama, Japan). At the start of each experiment, ca. 45 mM organic acids (i.e., lactate and acetate) were added as electron donors. Other details, including the reactor configuration, anolyte and catholyte composition, and temperature- and redox-condition maintenances are given in our previous works (Li et al., 2018). All the experiments in the present study were conducted at 35°C and pH 7. To know whether *S. decolorationis* NTOU1 is able to exploit mediators to boost EET/acetate degradation on a polarized electrode, four kinds of redox compounds, riboflavin, AQDS, Ru(NH_3_)_6_^3+^, and Fe(CN)_6_^3−^, were spiked into the electrochemical cell in separate experiments. These mediators were dissolved in the phosphate-buffer saline (i.e., PBS, consisted of 100 mM phosphate and 80 mM potassium chloride, adjusted to pH = 7) to generate the 0.2 and 0.4 mM dense solutions for the spiking processes, and an array of concentrations were consequently generated as listed in the Figure 3.

### Organic-Acid and Electrochemical Analyses

The organic acids in the anode medium were determined using a high-performance liquid chromatography (HPLC) equipped with a gradient pump (5160, Hitachi, Tokyo, Japan), a UV-Vis detector (5420, Hitachi, Tokyo, Japan), an autosampler (5280, Hitachi, Tokyo, Japan), and an ICSep COREGEL-87H3 column (7.8 mm × 300 mm) placed in an oven (Super CO-150, Enshine, Taipei, Taiwan) at 45°C. Sulfuric acid (8 mM) was used as a mobile phase at a flow rate of 0.5 mL min^−1^, and all the organic acids were detected at a wavelength of 210 nm. To better understand the steady-state catalytic current performance at different potentials, serial short-term chronoamperometry was carried out by manually adjusting the potential outputs on the Hokuto-Denko potentiostat. Each data point shown in Figure 4 was processed by waiting for 1 h at each potential and averaging the current data recorded in the last 40 min. All the potentials stated in this paper refer to the Standard Hydrogen Electrode.

### Scanning Electronic Microscope (SEM) Observation

The specimens (i.e., microbial cells attached on the graphite fibers) were fixed in a solution of 2.5% glutaraldehyde and phosphate buffer saline (100 mM phosphate and 80 mM KCl, pH 7.8, PBS) for 16 h at 4°C. Specimens were rinsed three times in the PBS to remove residual glutaraldehyde, for 10 min each time. Dehydration was carried out with a series of gradually increasing ethanol concentrations: 50, 75, 85, 95, and 100%. Finally, the dehydrated specimens were dried using the critical point drier (HCP-H2, Hitachi, Tokyo, Japan) and then coated with gold by ion sputtering (E101, Hitachi, Tokyo, Japan). Desiccated samples were observed using a TM3000 SEM (Hitachi, Tokyo, Japan).

### Mediators and Inhibitor Used in the Present Study

In the experiments described in Sections 3-2 and 3-4, the mediators and metabolic inhibitor were added to the electrochemical cells to clarify their effect on current generation and substrate degradation, namely, riboflavin (Sigma Chemical Co., United States), anthraquinone-2,6-disulfonate (AQDS, Tokyo Chemical Industry Co., Ltd., Japan), Ru(NH_3_)_6_^3+^ (Sigma Chemical Co., United States), Fe(CN)6^3−^ (Kanto Chemical Co., Inc., Japan), and AcMet (Sigma Chemical Co., United States). In addition to the mediators, the AcMet was also dissolved in the aforementioned PBS to generate a 1.2 M dense solution for the spiking processes, and an array of AcMet concentrations were consequently generated as listed in the Figure 5.

### Electron-Balance Calculations

When carrying out EET on an anode, *Shewanella* cells catalyze the substrate oxidation and electrode reduction, which also play a role as products (i.e., cell synthesis) of the entire reaction. In our recent study (Li et al., 2018), we developed a mathematic calculation describing cell growth, lactate consumption, as well as pyruvate and acetate production, which is based on the known central metabolic pathways involved in *S. oneidensis* MR-1 (Scott and Nealson, 1994). According to our previous findings, it is clarified that the intermediate (i.e., pyruvate and acetate) productions can indicate whether the lactate has been partially or completely oxidized, which are the major sources generating nicotinamide-adenine-dinucleotide equivalents to contribute current generation. To continuously track cell growth during the batch tests, the use of the mass-balance calculation, instead of destructive methods like protein assay, was proposed. By analyzing the organic-acid data detected using HPLC, the following electron-equivalent balance was developed to calculate biomass production:

(1) $$Q_{\text{Cell}}=-(12F\Delta n_{\text{La}}+10F\Delta n_{\text{Py}}+8F\Delta n_{\text{Ac}}+Q_{\text{EL}})$$

where *Q*_Cell_ is the metabolic charge used for biomass growth (not actually measured in this study); Δ*n*_La_ (<0), Δ*n*_Py_ (>0), and Δ*n*_Ac_ (>0) are the changes in the mole numbers of lactate, pyruvate, and acetate, respectively; *Q*_EL_ is the total charge of the electricity production calculated by integrating the electric current over time (i.e., ∫*i*d*t*); and *F* is the Faraday constant. The coefficients 12 (for lactate), 10 (for pyruvate), and 8 (for acetate) indicate the electron equivalents of biomass, lactate, pyruvate, and acetate, respectively. As for the experimental condition using acetate as the electron donor, Eq. (1) can be, respectively, rearranged as:

(2) $$Q_{\text{Cell}}=-(8\Delta n_{\text{Ac}}+Q_{\text{EL}})$$

**FIGURE 1:**
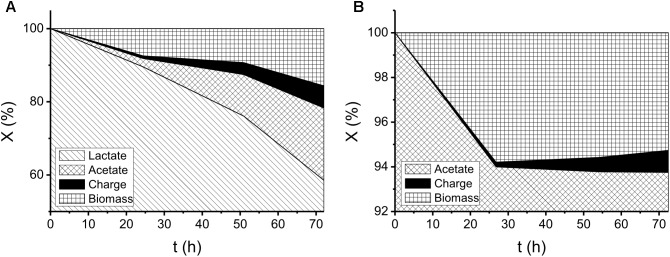
Profiles of electron distribution illustrating organic-acid metabolism for 72 h in the electrochemical cells using ca. 35 mM of **(A)** lactate and **(B)** acetate as electron donors. *X* in the *y* axis denotes the electron quantities as fractions derived from the total charge in the initial-substrate mass (i.e., 12*Fn*_La_ and 8*Fn*_Ac_ in **(A)** and **(B)**, respectively).

Some assumptions need to be made prior to validating Eq. (2): (i) the metabolism flows in the direction of acetate first converting to acetyl-CoA, then entering the TCA cycle; (ii) all biomass formation was assumed to proceed via the tricarboxylic acid (TCA) cycle; (iii) no unexpected intermediate was produced (e.g., TCA cycle intermediates) during the metabolism. To express the electron distribution shown in Figure 1, the electron-conversion (i.e., −12*F*Δ*n*_La_, 10*F*Δ*n*_Py_, 8*F*Δ*n*_Ac_, *Q*_Cell_, and *Q*_EL_) proportion is calculated over the initial electron pool (i.e., 12*Fn*_La_ and 8*Fn*_Ac_ at *t* = 0 h), in terms of percentage.

## Results

### *S. decolorationis* NTOU1 Utilizing Lactate and Acetate to Generate Current

**FIGURE 2:**
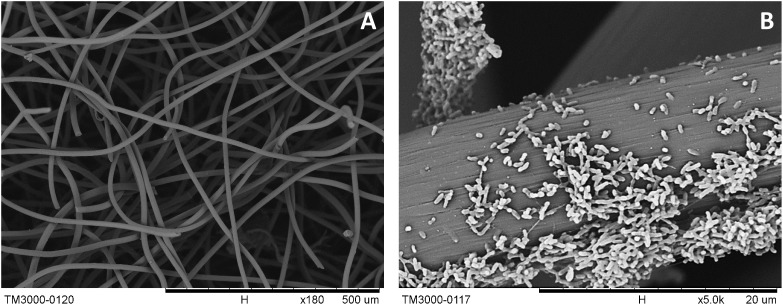
SEM images of *S. decolorationis* NTOU1 colonizing on the carbon-felt electrodes. **(A)** the clear structures of piled graphite fibers; **(B)** developed biofilms of *S. decolorationis* NTOU1 fed with acetate in the electrochemical cell under the poised potential of +630 mV.

During the batch experiments, no significant changes in pH were found over time. The electron distributions of different substrate utilization and intermediate production are shown in Figure 1. In the lactate-degrading experiment, 41% of the substrate was consumed after 72 h. Within the end product composition, 20, 6, and 16% of electrons were distributed to acetate, charge, and biomass, respectively. In the acetate-degrading experiment, only 6% of the substrate was consumed. It is noteworthy that at 28 h (Figure 1B), 6 and 0.2% of the electron were distributed to the biomass and charge production, respectively, but at 72 h, the proportions of them became 5 and 1%. The changing proportions indicated that the current generation from acetate was not perfectly growth associated, and presumably some cellular materials were lysed to generate current due to the decreasing of biomass-charge proportion (i.e., from 6 to 5%). To investigate the morphologies of the *S. decolorationis* NTOU1 grown on the carbon felt, the specimens for SEM observations were prepared after 31 h of electrochemical culturing. The SEM micrographs in the Figure 2A shows the clear structure of graphite fibers piling together in the carbon felt, and the Figure 2B shows the microbial cells colonizing on the graphite fibers with visible bacterial-nanowire-like morphologies, indicating that *S. decolorationis* NTOU1 was successfully cultivated on the electrode surfaces.

### The Effect of Different-Mediator Spiking on Current Generation and Acetate Degradation

**FIGURE 3:**
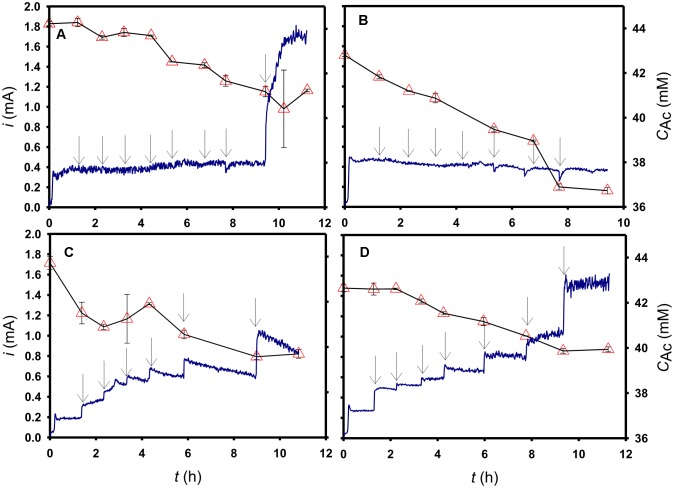
Time course profiles of the electric current (sold line) and the acetate concentration (open triangle) obtained with different mediator spikings: **(A)** riboflavin, **(B)** AQDS, **(C)** Ru(NH_3_)_6_^3+^, and **(D)** Fe(CN)_6_^3−^ spiked during the chronoamperometric processes. The arrows in **(A)** indicate the 0.5, 1, 2, 4, 8, 12, and 22 μM riboflavin, and 100 μM Fe(CN)_6_^3−^ in turn in the electrochemical cell; the arrows in **(B)** and **(D),** respectively, indicate the AQDS and Fe(CN)_6_^3−^ concentrations of 0.5, 1, 2, 4, 8, 12, and 22 μM in turn; the arrows in **(C)** indicate the Ru(NH_3_)_6_^3+^ concentrations of 0.5, 1, 2, 4, 8, and 18 μM in turn.

As shown in Figure 3A, with a series of riboflavin spiking (from 0.5 to 22 μM) the response current was not affected, but instead put out a steady current of ca. 0.44 mA after 1 h. After 22 μM riboflavin was entirely spiked into the electrochemical cell, a 100 μM Fe(CN)_6_^3−^ was spiked at *t* = 9.5 h, consequently resulting in a drastic current increase to 1.7 mA. From 0 to 9.5 h, acetate decreased from 44 to 41 mM, but after Fe(CN)_6_^3−^ spiking, the acetate concentration remained at 41 mM from 9.5 to 11.2 h without significant change. Similarly, no significant current change was found in the AQDS spiking experiment (Figure 3B). The acetate degrading rate was not affected before 8 μM AQDS spiking was done (i.e., at *t* = 5.3 h), but an unexpected acetate-concentration drop (i.e., from 39 to 37 mM) happened instantly after 12 μM AQDS spiking from *t* = 6.8 to 7.7 h. In Figure 3C,D, the response current immediately jumped on each spiking, resulting in staircase-like increments. After the spiking was complete, the final concentrations of Ru(NH_3_)_6_^3+^ and Fe(CN)_6_^3−^ both accumulated to 22 μM, and consequently their anodic currents reached 0.8 and 1.5 mA, respectively. With regard to the substrate concentration, the acetate degradation was found to stop at the last spike (i.e., 18 μM Ru(NH_3_)_6_^3+^ and 22 μM Fe(CN)_6_^3−^, respectively), although the currents at the same moment were found to be higher than prior performance in the same experiments.

### Electrochemical Characteristics of *S. decolorationis* NTOU1 Utilizing Acetate With/Without Mediator Additions

**FIGURE 4:**
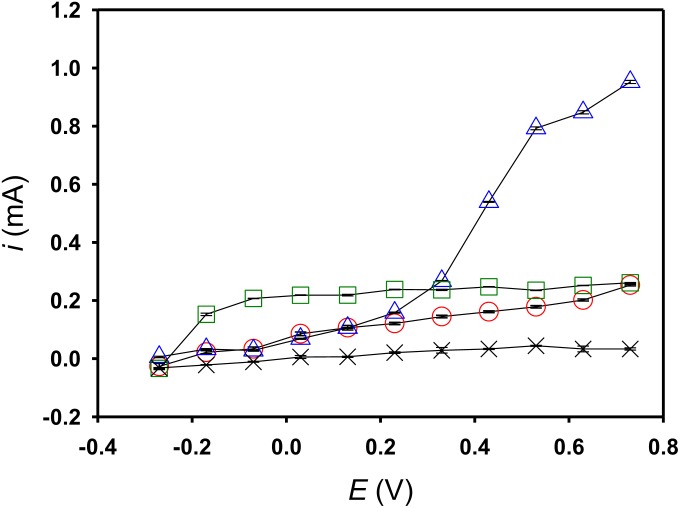
The steady-state catalytic currents of the acetate degradation recorded at different applied potentials. Open circle, *S. decoloration* NTOU1 digesting acetate without any mediator addition; open square, with 10 μM riboflavin addition; open triangle, with 10 μM ferricyanide addition; cross, only the acetate anolyte but without any mediator addition and *S. decoloration* NTOU1 inculation.

To plot the *i*-*V* graph, chronoamperometry (with +0.63 V poised) commenced by implementing a mediator-free running for ca. 20 h after inoculating *S. decolorationis* NTOU1. After 20 h, 10 μM riboflavin or Fe(CN)_6_^3−^ was spiked into each electrochemical cell, and the running was extended for an additional 2 h. After the aforementioned processes were complete, the potentials were manually adjusted to different levels and the responded current was recorded and averaged as described in the Section 2.3. To implement a mediator-control experiment, all the procedures were repeated, but this time mediator spiking was omitted. By reading the steady-state current increment in the same direction of the increasing poised potential, the catalytic current taking place at an onset of −0.27 V was found in all the experimental conditions (Figure 4). In the riboflavin-spiking experiment, the steady-state current was saturating at 0.07 V, but with regard to the mediator-control experiment, the steady-state current continued increasing from −0.27 to 0.73 V with no obvious saturation observed. In the Fe(CN)_6_^3−^-spiking experiment, a secondary catalytic current started at 0.13 V was found, in addition to the one starting at −0.27 V.

### Inhibiting Current Generation by Spiking AcMet

**FIGURE 5:**
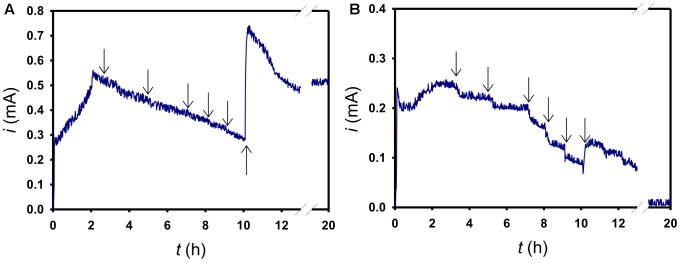
The effects of metabolic inhibitors on the current genration of *S. decolorationis* NTOU1 utilizing **(A)** lactate and **(B)** acetate. The arrows in both figures indicate the 6, 12, 24, 36, 48 mM AcMet, and 6 μM riboflavin in turn in the electrochemical cell.

To evaluate the role of c-type cytochromes in current generation, AcMet was spiked in an order of different concentrations, binding to the c-type cytochromes and rendering them non-functional (Nakamura et al., 2009). First, 6 mM of AcMet was added after 3.3 h of operation at +0.63 V; subsequently, each addition of AcMet was conducted every 1 or 2 h. In the lactate experiment (Figure 5A), the response current decreased after the first spiking. As the AcMet concentration increased from 6 to 48 mM, the rate of current decrease did not change significantly. After 48 mM AcMet were entirely added, 6 μM riboflavin was subsequently added into the electrochemical cell, which immediately rescued the current to ca. 0.73 mA following a mild reduction to ca 0.50 mA at 20 h. In the acetate experiment (Figure 5B), as the AcMet concentration increased from 6 to 48 mM, the current significantly decreased after each spiking. With the same procedure of adding 6 μM riboflavin, although the current could be immediately rescued from 0.07 to 0.12 mA at 10 h, the response current continued decreasing until total elimination at 20 h.

## Discussion

According to our preliminary work, we set a current-generating competition between *S. decolorationis* NTOU1 and *S. putrefaciens* ATCC8071 using thicker carbon felts (SG-224K, Osaka Gas, Osaka, Japan, cut into 1.4 × 5.5 × 18 cm^3^) and 35 mM lactate as a substrate, but the same cell configuration and electrolyte ingredients mentioned in Section 2-2; and the current generation results are shown in the Supplementary Figure S1. The major advantages of using *S. decolorationis* NTOU1 over *S. putrefaciens* ATCC8071 are a higher reaction rate and superior adaptability: after inoculating the same quantities of microbial cells, the experiment with *S. decolorationis* NTOU1 presented an immediate current generation, whereas there was a 40-h lag phase found when using *S. putrefaciens* ATCC8071. Regarding the performance of the current generation, the charge production of *S. putrefaciens* ATCC8021 (724 C) are lower than that of *S. decolorationis* NTOU1 (770 C). Combining the other performances including faster lactate degradation rate and greater coulomb efficiency (see the descriptions in the Supplementary Materials), it is indicated that *S. decolorationis* NTOU1 demonstrates superior performance to *S. putrefaciens* ATCC8071 when lactate was provided as an electron donor.

With our results suggesting that lactate is the preferable substrate (41% electron consumption) for EET, deliberately using acetate here becomes an alternative method to render *S. decolorationis* NTOU1 a “weak electricigen” that should be studied to understand what drives EET in response to electron-donor- or acceptor-limiting condition (Doyle and Marsili, 2018). In a separated experiment, we inoculated *S. oneidensis* MR-1 in the electrochemical cell and subsequently analyzed its current generation and acetate consumption. According to our results (Supplementary Figure S2), *S. oneidensis* MR-1 can hardly utilize acetate in 48 h, but a small amount of current could still be generated in the absence of acetate addition, indicating that EET-capable microorganisms may somehow utilizing their own cellular materials to implement EET (Freguia et al., 2007). To understand how many coulombs of charge could be produced via cell lysis of *S. decolorationis* NTOU1, the control experiments with/without acetate addition were implemented and the results are shown in the Supplementary Figure S3. By comparing the current-generating results, the charge production in the experiment of with acetate addition (i.e., 6 C in 16.8 h) was obviously higher than the one without acetate addition (i.e., 16 C in 16.8 h), indicating that a significant number of charge could be produced via acetate degradation. For acetate, only 6% of the substrate was removed, indicating that acetate was slightly utilized by *Shewanella* under anaerobic condition in the electrochemical cell. Although the acetate-degrading quantity is significant, this result agrees with those reported in other studies, which found similar acetate-accumulation (Park and Zeikus, 2002; Marsili et al., 2008). Considering the metabolic pathway, one mole of ATP is generated when one mole of acetyl-CoA is converted to one mole of acetate; vice versa, one mole of ATP is consumed when one mole of acetate is converted to acetyl-CoA with the aid of phosphotransacetylase, acetate kinase, and acetyl-CoA synthase (Kumari et al., 2000). When acetate is used as the sole substrate, unlike the lactate-utilizing conditions, there is no substrate-level phosphorylation pathway that may instantly generate sufficient ATPs to boost the catabolism of acetyl-CoA. Notably, an insufficient ATP level might impair the efficiency of current generation due to numerous ATPs being required to generate redox compounds such as riboflavin (Bacher et al., 2000; Dauner et al., 2002).

Fe(CN_6_)^3−^ possesses a higher formal potential (+0.36 V) than riboflavin (−0.3 V), and as such can better receive electrons from OMC or NDH of *Shewanella* (Li et al., 2018), thus driving a faster kinetics so as to generate a stronger current. Furthermore, the riboflavin is a proton-associated redox compound (though metal complexes such as Fe(CN_6_)^3−^ are not) that might absorb the protons available in the periplasm during EET, leading to less ATP production (i.e., turning down the oxidative phosphorylation) and unfavorable acetate degradation. To better clarify the effects of mediator potential and proton-absorbing characteristics, AQDS and Ru(NH3)_6_^3+^ were selected for further examination. Although the formal-potential difference between AQDS (−0.16 V at pH = 7, 25 C, Li et al., 2018) and Ru(NH3)_6_^3+^ (+0.12 V, Loew et al., 2017) is much smaller than the one between riboflavin and Fe(CN_6_)^3−^, AQDS also holds the characteristic of proton absorption during redox reactions. Therefore, it cannot enhance the current generation for the same reason as when riboflavin is used as a mediator. In addition, it was unexpectedly found that the acetate consumption rate slowed down even when the current got stronger with the Fe(CN_6_)^3−^ (Figure 3A), Ru(NH3)_6_^3+^ (Figure 3C), and Fe(CN_6_)^3−^ (Figure 3C) concentrations reading 100, 18, and 22 μM, respectively. It is presumably due to an unknown mechanism inhibiting acetate metabolism by metal-complex mediators; consequently, the cellular material of *S. decolorationis* NTOU1 was used for current generation.

While cyclic voltammetry (CV) is a potentiodynamic method which ramps potential linearly versus time in a quiescent solution, the advantage of carrying out the serial short-term chronoamperometry (mentioned in the Section 2.3) is that the non-faradaic current is much smaller than that observed in CV and that the catalytic current can be easily measured under the steadily mixing conditions. Since it is critical to exhibit the sigmoidal *i*-*V* curves that directly indicate which mediator is exactly carrying out turnover (i.e., activated microbial cells keep reducing mediators oxidized by the anodic electrode), we would suggest that in the case of utilizing less preferable electron donors (e.g., acetate in the present study), serial short-term chronoamperometry is a rather promising method that could better analyze the electrochemical characteristics of EET-capable microorganisms. In our past study, it was reported that *S. decolorationis* NTOU1 is able to secrete riboflavin and menaquinone on cell surfaces that are able to drive the catalytic current under the anaerobic condition (Li et al., 2010). The results shown in Figure 3A, 4 consistently indicate that with the 10 μM riboflavin addition, the current would not be enhanced at +0.63 V. Moreover, unlike the mediator-control experiment (i.e., open-circle symbols in the Figure 4), the steady-state current in the riboflavin-addition experiment (i.e., open-square symbols) did not ramp up from −0.07 to +0.73 V. This result indicates that the riboflavin addition might possibly inhibit some primitive mediator (e.g., menaquinone, −0.75 V, Hernandez and Newman, 2001) secretion by either turning down some specific genes (e.g., *menC* decoding menaquinone-synthesizing proteins, Newman and Kolter, 2000), or impairing mediator diffusion to the cell exterior. When the lactate is used as the substrate, it is found that *S. decolorationis* NTOU1 is less sensitive to AcMet (Figure 5A). This result agrees with a recent study using potassium cyanide as an OMC inhibitor of *Shewanella* strain Hac319 (Takeuchi et al., 2018). The EET activities rescued by diffusive-riboflavin addition (Figure 5) indicate that in addition to the model of flavin-bound OMC (aka flavocytochrome, Edwards et al., 2015), diffusive riboflavin can still assist EET after a large fraction of OMCs been deactivated. In the present study, the interactions between primitive mediators and OMCs are not yet clear. However, when acetate is used as the substrate, the significant current response to AcMet indicates the importance of OMC acting as the last step of oxidative phosphorylation, the only pathway to phosphorylate ADP to ATP during acetate metabolism.

## Conclusion

To clarify the status of EET when *S. decolorationis* NTOU1 simultaneously exploits acetate and externally added mediators, this study is comprised four individual experiments which concurrently provide strong evidences by comparing different substrate utilization, mediator potentials and chemical characteristics. The combination of electrochemical and instrumental analyses used in this work leads to the following conclusions:

1. *Shewanella decolorationis* NTOU1 is holding significant acetate-exploiting capabilities for EET, although acetate is considered as a less preferable substrate for *S. decolorationis* NTOU1 (only 6% electron consumed after a 72-h incubation).
2. Considering the cross-comparison results based on the formal potentials and chemical characteristics of the four externally added mediators used in this study, it is apparent that the proton-associated mediators (i.e., riboflavin and AQDS) do not significantly assist current generation, but the metal-complex mediators (i.e., Fe(CN)_6_^3−^ and Ru(NH_3_)_6_^3+^) do, when acetate is used as a substrate.
3. According to the electrochemical analyses, the results show that the riboflavin addition affects primitive mediator production, but Fe(CN)_6_^3−^ does not. The inhibitor adding experiment also reveals the crucial role of OMCs when *S. decolorationis* NTOU1 exploits acetate for EET.

## Author Contributions

S-LL conceived and designed the experiments. S-LL, Y-JW, and Y-CC performed the experiments. S-LL and C-PY wrote the manuscript. S-ML and C-PY analyzed the data. S-ML provided the pure strains of *Shewanella* spp.

## Conflict of Interest Statement

The authors declare that the research was conducted in the absence of any commercial or financial relationships that could be construed as a potential conflict of interest.
