# Supplementary material for: Chemical Characteristics of Electron Shuttles Affect Extracellular Electron Transfer: Shewanella decolorationis NTOU1 Simultaneously Exploiting Acetate and Mediators
Source: Front Microbiol. 2019 Mar 5;10:399. doi: 10.3389/fmicb.2019.00399 (PMC6411715; doi:10.3389/fmicb.2019.00399)
Supplement: Supplementary file 1 [file Data_Sheet_1.docx]

Supplementary Material

Chemical Characteristics of Electron Shuttles Affect Extracellular Electron Transfer: *Shewanella* *decolorationis* NTOU1 Simultaneously Exploiting Acetate and Mediators

Shiue-Lin Li^1^, Yu-Jie Wang^1^, Yu-Chun Chen^1^, Shiu-Mei Liu^2^, Chang-Ping Yu^1,*^

*** Correspondence:** Corresponding Author: [cpyu@ntu.edu.tw](mailto:cpyu@ntu.edu.tw)

# Supplementary Figures

**Comparison of the electricity-generation capability capabilities between *S. decolorationis* NTOU1 and *S. putrefaciens* ATCC 8071**

A batch test was carried out to compare two *Shewanella* strains, *S. decolorationis* NTOU1 and *S. putrefaciens* ATCC8071, using an electrochemical cell with thick carbon felt. The batch experiments was lasted until the current generation ceased. In the experiment with *S. decolorationis* NTOU1, the maximum current was obtained ca. 100 A m^−3^ and the electricity production completely stopped after 60 h. During the 72-h experiment, 770 C of the charge was produced with a change in lactate concentration from 39 to 2.4 mM. In addition to the charge production, 5 mM of pyruvate and 23 mM of acetate were also produced. In the experiment with *S. putrefaciens* ATCC 8071, a 40-h retardation of the current generation was observed; a maximum current of ca. 70 A m^−3^ was achieved at 83 h. The current generation ceased after 100 h. During the 120-h experiment, 724 C of charge was produced with concomitant production of 29 mM acetate and 12 mM formate, but no obvious pyruvate production. The lactate was degraded from 39 to 0.2 mM for a removal ratio of 99.5%. Columbic efficiencies (evaluated by calculating −(*Q*_EL_/12*F*Δ*n*_La_) × 100%) of 18 and 16% were obtained in the experiments with *S. decolorationis* NTOU1 and *S. putrefaciens* ATCC8071, respectively.

##### Supplementary Figure S1. Current production in an electrochemical cell using *S. decolorationis* NTOU1 and *S. putrefaciens* ATCC8071. The experiments were conducted at 30 ^o^C with ca. 39 mM of lactate added to the anode medium. Blue line, current production of *S. decolorationis* NTOU1; red line, current production of *S. putrefaciens* ATCC8071; open square, lactate degradation of *S. decolorationis* NTOU1; open triangle, lactate degradation of *S. putrefaciens* ATCC8071.

##### Supplementary Figure S2. Profiles of current generation and acetate consumption in an electrochemical cell inoculated with *S. oneidensis* MR-1. Red line and red open circle, data responding to the 45-mM-acetate initial condition; blue line and blue open circle, 15 mM acetate; black line and black open circle, 0 mM acetate.

**Supplementary Figure S3.** Responded current on the carbon-felt electrode poised at +0.63 V. Red line: *S. decoloraitonis* NTOU1 with 45 mM acetate as electron donor; blue line, *S. decoloraitonis* NTOU1 without any external electron donor.
